# Altered Gut Microbiome in Parkinson’s Disease and the Influence of Lipopolysaccharide in a Human α-Synuclein Over-Expressing Mouse Model

**DOI:** 10.3389/fnins.2019.00839

**Published:** 2019-08-07

**Authors:** Anastazja M. Gorecki, Leah Preskey, Megan C. Bakeberg, Jade E. Kenna, Christi Gildenhuys, Gabriella MacDougall, Sarah A. Dunlop, Frank L. Mastaglia, P. Anthony Akkari, Frank Koengten, Ryan S. Anderton

**Affiliations:** ^1^Perron Institute for Neurological and Translational Science, Nedlands, WA, Australia; ^2^Centre for Neuromuscular & Neurological Disorders, The University of Western Australia, Crawley, WA, Australia; ^3^Ozgene Pty Ltd., Bentley, WA, Australia; ^4^School of Medicine, The University of Notre Dame Australia, Fremantle, WA, Australia; ^5^Institute for Health Research and School of Health Sciences, The University of Notre Dame Australia, Fremantle, WA, Australia; ^6^School of Biological Sciences, The University of Western Australia, Nedlands, WA, Australia; ^7^The Centre for Molecular Medicine and Innovative Therapeutics, Murdoch University, Murdoch, WA, Australia

**Keywords:** Parkinson’s disease, microbiome, lipopolysaccharide, Gammaproteobacteria, Thy1-αSyn, gastrointestinal

## Abstract

The interaction between the gut microbiota and alpha-synuclein (αSyn) aggregation in Parkinson’s disease (PD) is receiving increasing attention. The objective of this study was to investigate gut microbiota, and effects of an inflammatory lipopolysaccharide (LPS) trigger in a human αSyn over-expressing mouse model of PD (Thy1-αSyn). Stool samples from patients with confirmed PD and Thy1-αSyn mice were analyzed using 16S ribosomal RNA sequencing. Compared to healthy controls, the relative abundance of mucin-degrading Verrucomicrobiae and LPS-producing Gammaproteobacteria were greater in PD patients. In mice, the abundance of Gammaproteobacteria was negligible in both Thy1-αSyn and wild-type (WT) animals, while Verrucomicrobiae were reduced in Thy1-αSyn mice. The effect of LPS on intestinal barrier function was investigated *in vitro* using intestinal epithelial (IEC-6) cells, and *in vivo* via administration of LPS in drinking water to Thy1-αSyn mice. Acute exposure to LPS *in vitro* resulted in a reduction and altered distribution of the tight junction markers ZO-1 and e-Cadherin around the cell membrane in IEC-6 cells, as shown by immunohistochemistry. LPS administration in Thy1-αSyn mice resulted in the emergence of early motor manifestations at 10 weeks, compared to untreated mice who were still asymptomatic at this age. This study reaffirms that an altered microbiome exists in patients with PD, and supports the notion of a proinflammatory gut microbiome environment as a trigger for PD pathogenesis.

## Introduction

Parkinson’s disease (PD) has traditionally been characterized by motor impairment but is now considered a multisystemic disorder displaying a plethora of non-motor symptoms (Chaudhuri et al., 2006; Emamzadeh and Surguchov, 2018; Greenland et al., 2019). For example, people with PD frequently report various gastrointestinal complaints including constipation and nausea, and have prolonged intestinal transit time, often years prior to their PD diagnosis (Martinez-Martin et al., 2011; Lin et al., 2014; Fasano et al., 2015; Adams-Carr et al., 2016). A key feature of the disease is the formation of insoluble alpha-synuclein (αSyn) aggregates within neurons (Goedert et al., 2013), contributing to the loss of dopaminergic neurons in the basal ganglia. This Lewy body pathology also occurs more widely throughout the central and peripheral nervous systems, including the enteric nervous system (Beach et al., 2010).

The concept that PD is initiated following continuous gut aggravation has gathered significant momentum in recent years. Enteric αSyn is associated with greater intestinal permeability (Forsyth et al., 2011), and a positive relationship between inflammatory bowel diseases and future PD risk is evident in various populations (Lin et al., 2016; Peter et al., 2018; Weimers et al., 2018). Individuals with PD also exhibit an imbalanced gut microbiome (dysbiosis) and gastrointestinal inflammation (Keshavarzian et al., 2015; Hill-Burns et al., 2017; Heintz-Buschart et al., 2018). Various studies report similar trends in the microbial composition of people with PD, where commensal bacteria (e.g., phylum Firmicutes) are reduced, while pathogenic gram-negative bacteria (Proteobacteria, *Enterobacteriaceae*, *Escherichia* sp.) and mucin-degrading *Verrucomicrobiaceae* are increased (Keshavarzian et al., 2015; Scheperjans et al., 2015; Unger et al., 2016; Hill-Burns et al., 2017; Li et al., 2017). Moreover, bacterial treatments *in vitro* and fecal microbial transplants *in vivo* also support the role of the gut microbiome in αSyn aggregation, gastrointestinal inflammation and motor symptom development (Sampson et al., 2016; Choi et al., 2018; Sun et al., 2018).

Gram-negative bacteria, elevated in people with PD, produce lipopolysaccharide (LPS), an endotoxin associated with intestinal inflammation (Guo et al., 2015; Nighot et al., 2017). Interestingly, the abundance of gram-negative *Enterobacteriaceae* is positively correlated with the degree of postural instability and gait difficulty in individuals with PD (Scheperjans et al., 2015). In rodent models, LPS administration mirrors PD pathology. Direct stereotaxic injection of LPS into the substantia nigra causes microglial inflammation, oxidative stress, cellular apoptosis, reduced dopamine production and motor impairments (Sharma and Nehru, 2015). In the periphery, an intraperitoneal dose of LPS increased αSyn expression and intestinal permeability in the large intestine (Kelly et al., 2014), while chronic intranasal instillation resulted in progressive hypokinesia, selective dopaminergic neuronal loss and nigrostriatal αSyn aggregation (He et al., 2013). Recently, intrarectal administration of *Proteus mirabilis*-derived LPS to mice was shown to reduce the tight junction cell marker occludin but increased tumor necrosis factor alpha levels, and caused toll-like receptor 4 overexpression in the colon 16 days after treatment (Choi et al., 2018). These effects extended to the brain, with microglial activation throughout nigrostriatal regions and αSyn aggregation throughout central and enteric neurons, supporting evidence for environmentally-triggered gut-brain pathology in the context of PD (Choi et al., 2018). With enteric levels of αSyn being associated with greater intestinal permeability and LPS translocation across the intestinal barrier in people with PD (Forsyth et al., 2011), there is the potential for gut microbiota to induce αSyn propagation along peripheral nerves toward the brainstem, and brain more widely.

As such, early gastrointestinal dysfunction in people with PD may be more than a prodromal symptom, but rather an early contributing factor for αSyn pathology in susceptible individuals. Therefore, the objective of this study was to investigate the gut microbiota in PD patients and αSyn over-expressing mice. Mice over-expressing human αSyn (C57BL-6N-Tg (Thy1-SNCA) 61Mjff/J) exhibit nigrostriatal pathology and dopamine depletion (Chesselet et al., 2012), and were chosen for this study as a progressive model of PD which allows for a clinically relevant exploration of the gut microbiome prior to the onset of motor impairments. The identification of pro-inflammatory Gammaproteobacteria in PD patients but not the genetic rodent model led to the exploration of LPS as an environmental inflammatory trigger for PD in intestinal epithelial cells and αSyn over-expressing mice.

## Materials and Methods

### Human Participants

Participants were recruited from The Movement Disorders Clinics at the Perron Institute for Neurological and Translational Science. Patients (*n* = 14) and healthy controls (*n* = 7) with no history of antibiotic or non-steroidal anti-inflammatory drug use in the previous 3 months were included in this study. All patients were confirmed to have idiopathic Parkinson’s disease by a movement disorders neurologist, in accordance with the United Kingdom Brain Bank criteria. Human Research and Ethics approval was granted from The University of Western Australia (Approval number RA/4/20/4470). All participants gave written informed consent in accordance with the Declaration of Helsinki. Clinical and demographic characteristics of the participants recruited are presented in Supplementary Table S1.

### Participant Clinical Information and Fecal Sample Collection

Relevant participant demographic and clinical information was obtained from healthy controls and patients. At the time of all assessments, patient response to their dopaminergic medications was at optimum levels (“ON” period), and motor symptoms were evaluated using the MDS-Unified Parkinson’s Disease Rating Scale (MDS-UPDRS) Part III and Hoehn and Yahr Scale, as previously described (Evans et al., 2017). For subsequent analyses the patients were divided into mild (*n* = 7) and severe (*n* = 7) groups based on MDS-UPDRS PIII and Hoehn and Yahr scores. Rectal swab samples were used to collect stool samples for DNA extraction and 16S rRNA sequencing. Samples were obtained by inserting a dual Dacron swab moistened with sterile liquid Stuart medium (Becton Dickenson, Sparks, MD) 1–2 cm past the anal verge and rotating the swab gently through 360°. Collected swabs were stored in liquid Stuart medium at −20°C prior to DNA extraction.

### Wild-Type and Thy1-SNCA Mice

Mice overexpressing wild-type human αSyn (Thy1-SNCA) throughout the central and enteric nervous systems exhibit progressive αSyn pathology, nigrostriatal dopamine depletion and inflammation coupled with motor and non-motor symptoms, including slowed colonic transit time and constipation (Chesselet et al., 2012; Hallett et al., 2012). Four male hemizygous mice overexpressing wild type human α-Synuclein (C57BL-6N-Tg (Thy1-SNCA)15Mjff/J) (αSyn) were acquired from Jackson Laboratories (Jax Stock #017682) (Ouyang, 2012) and bred to female C57BL/6N mice to generate the male and female heterozygous offspring used in this study. Wild-type (WT) controls and αSyn littermates were housed together and received food and water *ab libitum*. All animals were maintained on a 12-hour light-dark cycle with constant temperature and humidity at the same facility. For animal studies, fecal pellets were collected from individual WT controls and αSyn mice at 8 weeks of age. All animal husbandry and experiments were approved by the Ozgene Animal Ethics Committee.

### Functional Assessments

#### Adhesive Removal

Small adhesive stickers (30 mm × 40 mm) were placed on the nasal bridge. Timing began once the mouse was released into the home cage. Time between first paw contact with the sticker and removal of the sticker was measured. The mean of all three replicates per day was used for further analysis (Guyenet et al., 2010).

#### Hind Limb Clasping Reflex

The mouse was suspended in the air by its tail, and the distance of the hind limbs to the abdomen for the majority of 10 s was scored on a 4-point scale, as previously reported (Sampson et al., 2016). A score of 0 was given if hind limbs are constantly splayed outward from the abdomen, a score of 1 if one hind limb was retracted toward abdomen for more than 50% of the time suspended, and two if both limbs are retracted. A maximum score of three indicates both hind limbs are entirely retracted and touching the abdomen for more than 50% of the suspended time (Guyenet et al., 2010). Retraction of the hind limbs during the tail suspension test is considered to be a dystonic type of reaction indicative of striatal dysfunction (Zhang et al., 2014; Sampson et al., 2016).

### DNA Extraction and 16S rRNA Sequencing

Human stool swabs and mouse fecal pellets were sent to the Australian Genome Research Facility (AGRF, Australia) for total nucleic acid extraction, 16S rRNA sequencing and microbial diversity mapping of the V3-V4 region, as previously described (Reyes et al., 2010). For this study, relative abundance and number of different Operational Taxonomic Units (OTU, diversity) were provided from the data sequencing.

### Small Intestinal Epithelial Cell Culture

Rat small intestinal epithelial cells (IEC-6; ECACC 88071401), a model for the crypt region which regulates intestinal permeability (Kimura et al., 1997) were supplied by CellBank Australia (WestMead, NSW, Australia). IEC-6 cells were maintained in Dulbecco’s Modified Eagle’s Medium (DMEM)/5% fetal calf serum (FCS; Thermo Fisher, Melbourne, Australia) containing penicillin (20 U/ml) and streptomycin (20 mg/ml) at 37°C in a CO_2_ incubator (5% CO_2_, 95% air balance, 97% humidity). For experimentation, IEC-6 cells were seeded in 24-well plates at approximately 5 × 10^4^ cells/well in 500 μl DMEM/5% FCS and used for studies 1–3 days after plating.

### Lipopolysaccharide Treatment

Both IEC-6 cells and mice were treated with LPS (E. coli 055: B5, Sigma, Australia). For *in vitro* studies, culture media was aspirated from each well and replaced with DMEM/5% FCS containing LPS (10 μg/ml final concentration) and maintained at 37°C in a CO_2_ incubator (5% CO_2_, 95% air balance, 97% humidity) for 4 h. For animal studies, animals were provided with drinking water containing LPS (5 μg/ml) diluted in 0.075 M sucrose. LPS was provided for 15 h starting at lights out (6:00 pm) for 10 consecutive days starting at 8 weeks of age. A low dose of oral LPS for 10 days was chosen to mimic clinically relevant chronic exposure to luminal LPS produced by gut microbes. Unlike systemic LPS administration, oral LPS does not cause severe neurological deficits (Suffredini et al., 1999; Inagawa et al., 2011).

### Tissue Preparation

Cervical dislocation and tissue harvesting were performed at various timepoints following behavioral assessments. Small and large intestines were flushed with PBS and optimal cutting temperature (OCT) compound (Tissue-Tek), and fresh-frozen in liquid nitrogen. Tissue was thawed to −20°C and subsequently sectioned onto glass slides using a cryostat microtome (CM1510, Leica, Germany) for immunofluorescence analysis.

### Protein Extraction and Western Blots Analysis

Western blot analysis was completed as previously described (MacDougall et al., 2017). Briefly, 15 mg of tissue homogenate from each sample was separated by sodium dodecyl sulfate-polyacrylamide gel electrophoresis (SDS-PAGE) using pre-cast Bis-Tris gels (10%; Bio-Rad). Membranes were blocked in PBS-Tween 20 (0.1%) with ovalbumin (1 mg/ml) for 1 h, followed by overnight incubation (4°C) with alpha-synuclein (1:1000; Abcam, 212184), human alpha-synuclein (1:1000; Invitrogen 701085), or β-tubulin (1:3000; Invitrogen MA1-118) diluted in 1% PBS-T, and 1-hour incubation at room temperature with goat anti-rabbit Star Bright blue 700 (1:5000; Bio-Rad 12004162) or goat anti-mouse IgG HRP (1:20000; Bio-Rad 1705047) secondary antibodies. All membranes were visualized using ChemiDoc Imaging System (Bio-Rad). Quantification and densitometry were performed using ImageJ software (National Institutes of Health, United States).

### Immunocytochemistry and Immunofluorescence

Mounted tissue sections or IEC-6 cells were fixed in a 1:1 solution of methanol/acetate and incubated at −20°C for 15 min. After aspiration of the fixative, IEC-6 cells and mounted tissue sections were air-dried for 30 min at room temperature. IEC-6 cells were then washed in PBS + 0.2% Tween, and then blocked in 10% bovine serum albumin (BSA) for 15 min. Tissue sections were gently rinsed with PBS and blocked in 10% goat serum/1% BSA in 0.05% Triton X-100 PBS for 2 h. IEC-6 cells and tissue sections were incubated with TLR4 (1:200; Thermo Fisher), ZO-1 (1:400; Thermo Fisher), e-Cadherin (1:400; Thermo Fisher) primary antibodies overnight at 4°C, rinsed and incubated with goat anti-rabbit Alexa Fluor 555 (1:400, Bio-Rad) secondary antibodies for 2 h at room temperature. Prior to the final PBS wash, nuclei were stained with DAPI 0.5 μg/ml (Sigma-Aldrich, St. Louis, MO, United States). Tissue sections and cells were imaged with a fluorescent microscope (Olympus IX70; Olympus DP70 digital camera; Olympus).

### Statistical Analysis

Statistical analysis was conducted through IBM-SPSS (v.24, IBM Corporation) and GraphPad Prism (version 7, GraphPad, Inc., La Jolla, CA, United States) software. Results are shown as mean (SD) unless otherwise indicated, with a *p* < 0.05 considered significant. PD patients were dichotomized into mild and severe groups by a specialized clinician. Extreme outliers in abundance data were excluded as determined through ROUT (regression and outlier removal) calculations (via GraphPad Prism). For densitometric analysis of tissue lysate blots, mean difference was determined via an independent *t*-test. Behavioral assessment differences were compared cross-sectionally using the Mann-Whitney *U* tests, and before and after LPS treatment using paired *t*-tests.

## Results

### Bacterial Diversity and Abundance Are Altered in Parkinson’s Disease

**FIGURE 1:**
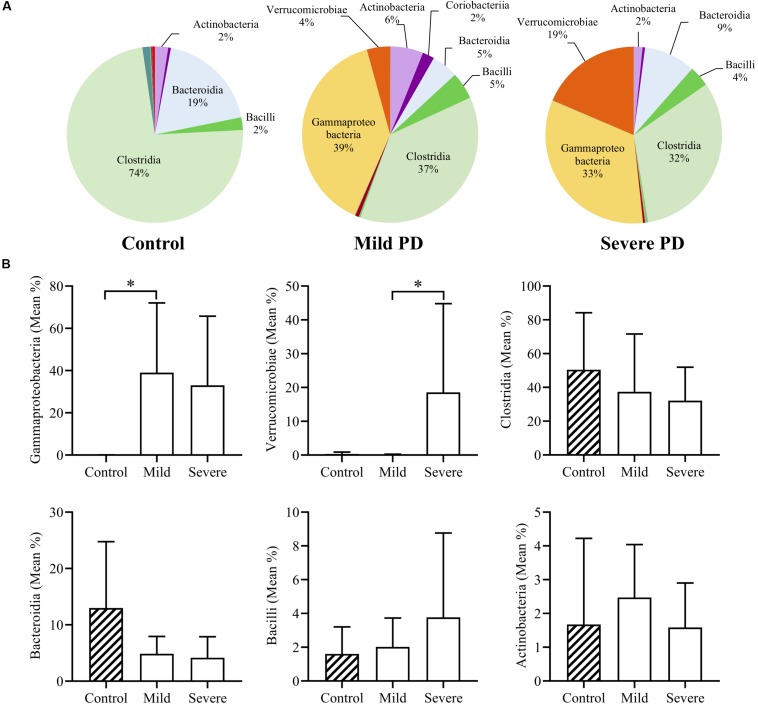
Mean class abundance in people with Parkinson’s disease and healthy controls. Individuals with Parkinson’s disease (PD) were grouped by a clinician. Pie charts illustrate the mean relative abundance of bacterial classes in healthy controls and individuals with mild or severe PD **(A)**. Comparison of the six most abundant classes demonstrates a significant increase in Gammaproteobacteria in mild PD compared to controls (621.22× fold, *p* < 0.05), and significantly elevated levels of Verrucomicrobiae in severe PD patients, when compared to mild PD patients (4.35× fold, *p* < 0.05) **(B)**.

Mean bacterial diversity based on total number of OTUs present, did not significantly differ between healthy control (236.10 ± 83.53) and PD patients (220.00 ± 76.05). After grouping by PD severity, the mean diversity of severe PD patients (174.90 ± 29.70) was significantly lower than that of mild patients (265.1 ± 83.03, *p* < 0.05). The four dominant phyla (Firmicutes, Bacteroidetes, Proteobacteria, and Actinobacteria) comprised 98.4% of mean control microbiota, and only 88.1% of mean PD microbiota. Mean bacterial class abundance is shown for controls, and mild and severe PD groups (Figure 1A). Notably, mean Clostridia abundance decreased with increasing disease severity, with a concomitant increase in classes Gammaproteobacteria and Verrucomicrobiae. The six most abundant bacterial classes were further investigated (Figure 1B). Both mild and severe PD groups demonstrated a higher mean abundance of Gammaproteobacteria than controls, with a significant increase in the mild PD group (621.22× fold, *p* < 0.05). Severe patients exhibited elevated levels of Verrucomicrobiae than controls, and a significant difference was evident when compared to the mild PD group (4.35× fold, *p* < 0.05). Clostridia and Bacteroidia were more abundant in healthy controls than mild and severe PD groups, but these differences did not reach statistical significance.

### Thy1-αSyn Over-Expressing Mice

**FIGURE 2:**
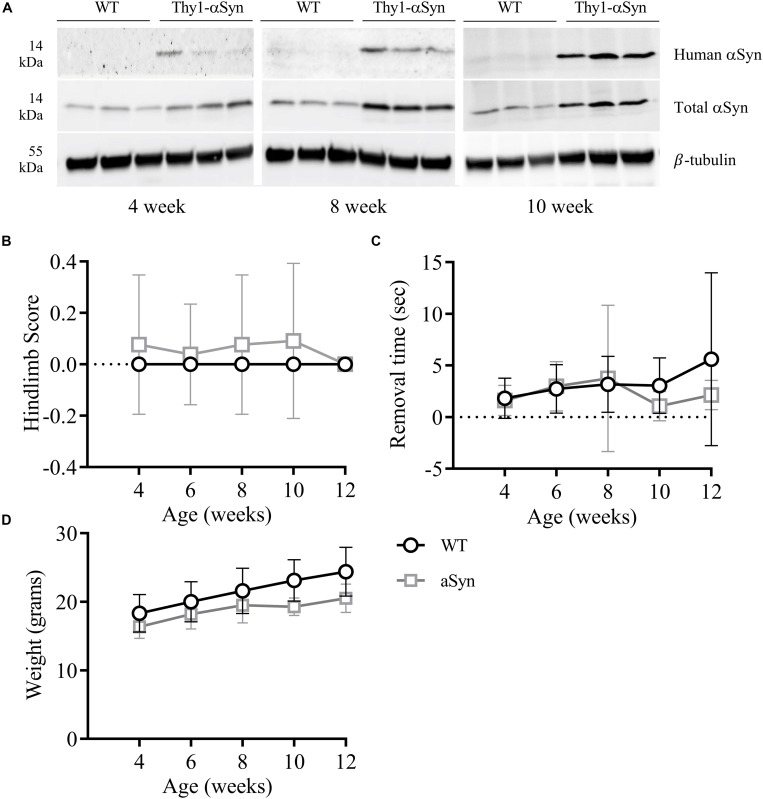
Progessively increasing brain αSyn levels are not associated with notable motor impairments in 12-week old mice. Compared to WT mice, the Thy1-αSyn mice exhibit progressively increasing human and total αSyn in whole brain lysates compared to WT mice at 4, 8 and 12 weeks of age **(A)**. There were no significant differences in hind-limb clasp scores **(B)** or nasal adhesive removal **(C)** between animal groups. The weight of both groups increased with age, where the Thy1-αSyn mice consistently weighed less **(D)**.

Whole brain homogenates and sections from Thy-1 αSyn-overexpressing (Thy1-αSyn) mice and wild-type littermates (WT) were analyzed for levels of αSyn at multiple time points. At 4, 8, and 12 weeks of age, human αSyn was detected in Thy1-αSyn mice but not the WT controls (Figure 2A). Interestingly, total αSyn protein progressively increased between time points, with a ∼1.5-fold increase in 4-week old, and ∼3 fold in 8 and 12-week old Thy1-αSyn brain tissue, when compared to WT controls. No significant differences in hindlimb clasp reflex (striatal dysfunction, Figure 2B) or nasal adhesive removal (fine motor control, Figure 2C) were observed from weeks 4–12 between Thy1-αSyn and WT mice. No differences in general locomotion or behavior were observed between the Thy1-αSyn and WT mice. The mean weight of Thy1-αSyn mice was lower than their WT littermates, with a mean weight 16% lower than WT controls at 12 weeks of age (Figure 2D and Supplementary Table S2).

### Bacterial Diversity and Abundance Are Altered in Thy1-αSyn Mice

**FIGURE 3:**
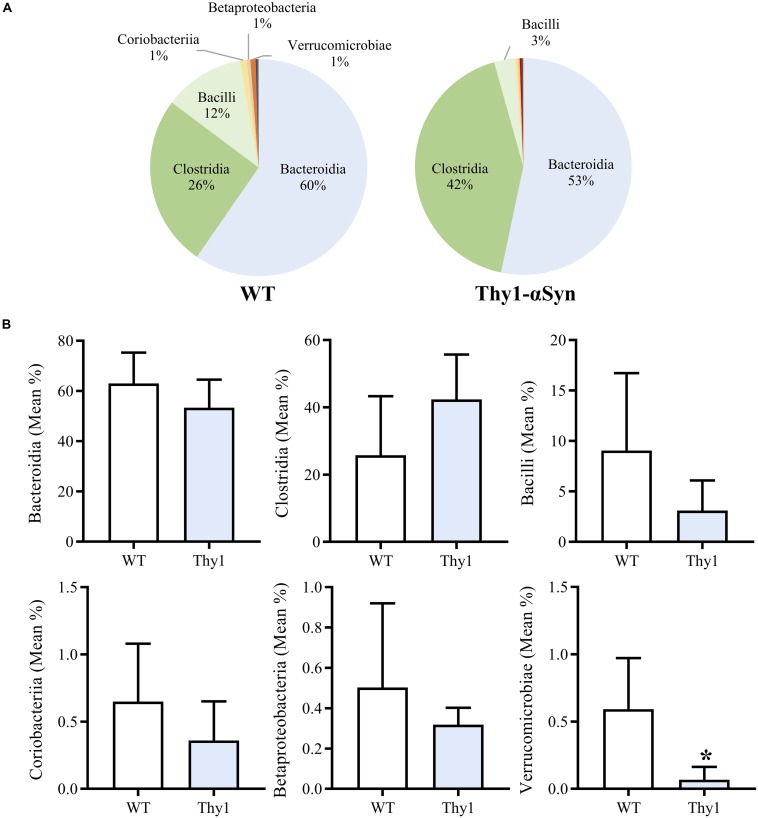
Thy1-αSyn mice do not demonstrate a pro-inflammatory gut microbiome at 8 weeks of age. Bacteroidia, Clostridia and Bacilli account for the majority of the microbiome in both WT and Thy1-αSyn mice as per 16S rRNA sequencing at 8 weeks of age **(A)**. Thy1-αSyn mice exhibit a significantly lower abundance of Verrucomicrobiae, however, the mean relative abundance of Bacteroidia, Clostridia, Bacilli, Coriobacteria and Betaproteobacteria are not significantly different **(B)**.

Thy1-αSyn and WT mice groups both showed a high mean microbial diversity, and Thy1-αSyn (347.6 ± 22.81) mice had a significantly greater diversity than WT controls (306.3 ± 21.55, *p* < 0.05). Overall classes Bacteroidia, Clostridia and Bacilli comprised the majority of the microbiome in both WT and Thy1-αSyn mice (Figure 3A). The six most abundant bacterial classes were compared between animal groups (Figure 3B). Verrucomicrobiae was significantly lower in Thy1-αSyn mice (mean relative abundance ± SD = 0.0007 ± 0.0009) than WT controls (0.0059 ± 0.0038, *p* < 0.05). However, the mean relative abundance of classes Bacteroidia, Clostridia, Bacilli, Coriobacteria and Betaproteobacteria were not significantly altered between Thy1-αSyn and WT mice.

### LPS Alters Quantity and Distribution of ZO-1 and e-Cadherin in IEC-6 Cells *in vitro*

**FIGURE 4:**
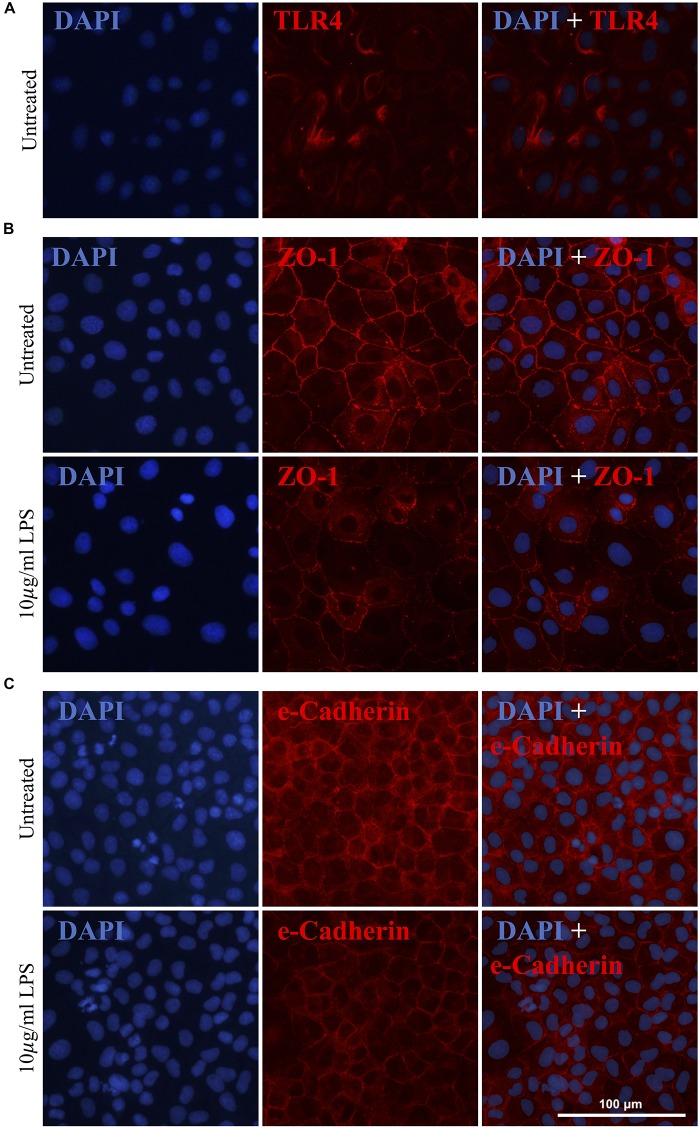
Gammaproteobacteria-produced LPS treatment increases intestinal permeability *in vitro*. IEC-6 cells express TLR4, a known LPS receptor **(A)**. Acute LPS treatment (10 μg/ml for 4 h) resulted in a noticeable reduction of and alteration in the distribution of ZO-1 **(B)** and e-Cadherin **(C)**.

As previously mentioned, gram-negative Gammaproteobacteria produce LPS, an inflammatory endotoxin. Given the increased abundance of Gammaproteobacteria in the pilot clinical cohort and the inferred increase in intestinal LPS, we next explored the effects of LPS on IEC-6 intestinal epithelial cells *in vitro*. The expression of the known LPS receptor, TLR4, was confirmed in IEC-6 cells (Figure 4A). Acute exposure of IEC-6 cells to LPS (10 μg/ml) did not affect cell viability or expression of TNF-α when compared to untreated cultures (Supplementary Figure S1). However, LPS treatment resulted in a noticeable reduction and altered distribution of the tight junction proteins ZO-1 (Figure 4B) and e-Cadherin in cells (Figure 4C) when compared to untreated IEC-6 cells.

### LPS Administration Leads to the Emergence of Motor Symptoms in Thy1-αSyn Mice

**FIGURE 5:**
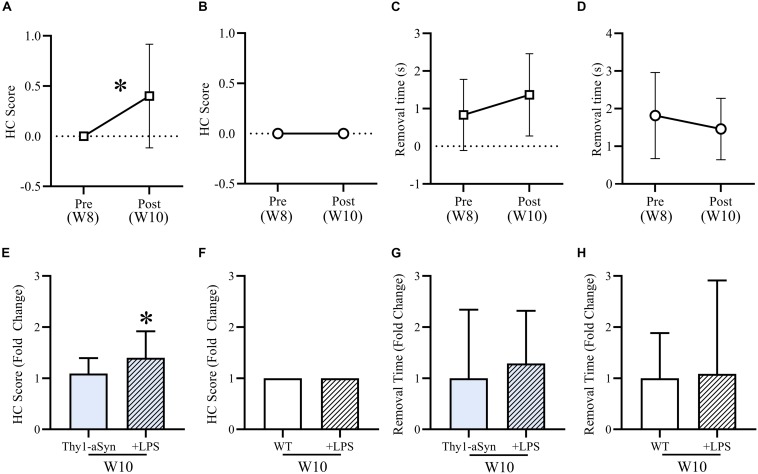
LPS treatment mildly exacerbates motor impairments in Thy1-αSyn mice. After LPS consumption (10 μg/ml) in drinking water for 12 nights, mean hindlimb clasp reflex (HC) score was significantly higher than baseline **(A)** and compared to untreated Thy1-αSyn mice **(E)**, while WT scores remained normal at all time points **(B,F)**. Mean adhesive removal time for Thy1-αSyn mice demonstrated a non-significant increase compared to baseline **(C)**, however, was lower than untreated 10-week-old Thy1-αSyn mice **(G)**. LPS-treated WT mice were non-significantly faster than baseline **(D)** and slightly slower than untreated animals at 10-week old **(H)**.

After LPS consumption (10 μg/ml) in drinking water for 12 nights, treated Thy1-αSyn mice had a significantly increased mean hindlimb clasp reflex (HC) score during the tail suspension test compared to baseline (Figure 5A) and untreated 10-week old Thy1-αSyn (Figure 5E), whose score remained normal (*p* < 0.05). WT groups maintained a normal HC score at all time points (Figures 5B,F). A non-significant trend for slower adhesive removal was evident when comparing LPS Thy1-αSyn mice (1.37s ± 1.09) to baseline (0.83s ± 0.95) (Figure 5C) and untreated 10-week old Thy1-αSyn mice (0.7s ± 0.81) (Figure 5G). LPS WT mice showed a non-significant trend for faster removal (1.82s ± 1.14) than baseline (3.29s ± 5.57) (Figure 5D) with no difference compared to untreated 10-week old WT (3.04s ± 2.69) (Figure 5H). After LPS consumption, Thy1-αSyn mice also displayed slower locomotion and altered behavior compared to baseline and WT animals.

Although it is unlikely to have impacted the observed motor impairments, both Thy1-αSyn and WT LPS-treated mice were significantly heavier than baseline. Treated Thy1-αSyn mice were also significantly heavier than untreated mice at 10-week old (Supplementary Figure S2). The observed weight gain is a possible consequence of the sucrose in the LPS mixture.

## Discussion

Previous studies have reported an altered gut microbiome and gastrointestinal inflammation in individuals with PD (Forsyth et al., 2011; Keshavarzian et al., 2015; Hill-Burns et al., 2017; Heintz-Buschart et al., 2018), however, it remains to be seen whether such changes are causative or symptomatic of the disease. Despite a small sample size, our data demonstrated a significant shift toward a dysbiotic gut microbiome in Australian people with PD, due to reduced diversity and altered relative class abundance. Subsequently, this study investigated the relationship between αSyn, motor impairments and microbial-derived LPS in an αSyn-overexpressing animal model of PD.

In contrast to patients with mild PD, those with more advanced disease were found to have significantly reduced bacterial diversity compared to healthy controls, in keeping with the findings of previous studies (Keshavarzian et al., 2015; Unger et al., 2016; Hill-Burns et al., 2017; Li et al., 2017). Lower bacterial diversity has been linked to intestinal inflammation and immune activation, and has also been reported in inflammatory bowel diseases such as Crohn’s disease and ulcerative colitis, as well as obesity (Ott et al., 2004; Eckburg et al., 2005; Ley et al., 2006; Manichanh et al., 2006; Frank et al., 2007; Dicksved et al., 2008; Carroll et al., 2011).

Our data demonstrated a significant increase in Gammaproteobacteria coupled with a non-significant reduction in Clostridia and Bacteroidia in people with PD. Clostridia and Bacteroidia are typically abundant throughout the healthy gut lumen (Lloyd-Price et al., 2016), and in this study were non-significantly reduced in mild and severe PD groups compared to healthy controls. Clostridia mostly consist of commensal bacteria located in the gastrointestinal mucosa, and genera including *Blautia, Faecalibacterium* and *Ruminococcus* are widely considered the principal producers of the anti-inflammatory metabolite butyrate (Sokol et al., 2008). *Clostridium tyrobutyricum* and various Bifidobacteria strains protect against LPS-induced inflammation and alterations to tight junction proteins *in vitro* (Riedel et al., 2006; Xiao et al., 2018). Interestingly, it has recently been reported that bacteria within Bacteroidia secrete an immunoinhibitory form of LPS which silences proinflammatory TLR4 signaling (d’Hennezel et al., 2017). Conversely, Gammaproteobacteria and Verrucomicrobiae, which normally represent only a small proportion of the healthy adult gut microbiome, are considered to be important for immune patterning and maintenance of mucin integrity (Eckburg et al., 2005). However, the abundance of Gammaproteobacteria is positively associated with metabolic disorders, inflammation and cancer, where increased Proteobacteria abundance is a proposed marker for dysbiosis (Shin et al., 2015). As a major contributor to LPS production, the finding of markedly elevated Gammaproteobacteria coupled with reduced Clostridia and Bacteroidia in both the mild and severe PD groups provides a basis for increased luminal LPS levels and reduced commensal, anti-inflammatory action in PD, which may impair intestinal integrity and cause chronic intestinal inflammation (Finnie et al., 1995; Lührs et al., 2002; Guo et al., 2015; Nighot et al., 2017). In low levels, *Akkermansia*, the only genus within Verrucomicrobiae, typically represent 1–4% of the gut microbiome and are beneficial mucin degraders that promote intestinal barrier turnover (Collado et al., 2007; Derrien et al., 2008). However, *Akkermansia muciniphila* exacerbates *Salmonella*-induced inflammation in gnotobiotic mice due to disruption of the protective mucus layer (Ganesh et al., 2013). Consequently, although both PD groups exhibited reduced commensal bacterial action, when coupled with high levels of Verrucomicrobiae in the severe PD group this may increase intestinal leakiness, facilitating translocation of luminal LPS to the enteric nervous system or systemic circulation. Systemic LPS has been linked to intestinal inflammation, which in turn is associated with enteric αSyn aggregation (Forsyth et al., 2011; Devos et al., 2013; Kelly et al., 2014). Alternatively, the elevated levels in severe patients may be a product of longer disease duration as Verrucomicrobiae thrive in low nutrient environments (Sonoyama et al., 2009). More thorough characterization of the gut microbiome, metabolomic profiling and intestinal protein expression is required in a larger sample size to clarify the influence of the gut microbiome on intestinal dysfunction in PD.

We subsequently examined if elevated CNS αSyn levels are associated with alterations in the gut microbiome prior to the onset of motor symptoms in Thy1-αSyn mice. Thy-1 promoter activity does not begin until postnatal day 10 (Chesselet et al., 2012; Hallett et al., 2012), which may underlie the progressive accumulation of human and total αSyn between 4 and 12 weeks of age, however, further studies are required to characterize αSyn levels throughout the brain and peripheral nervous system of Thy1-αSyn mice at different time points. Bacteroidia, Clostridia and Bacilli dominated the microbiome in both WT and Thy1-αSyn mice while Gammaproteobacteria abundance was very low, reflecting previous microbial profiling in healthy lab mice (Hildebrand et al., 2013). Antibiotic use, *Clostridium difficile*-associated colitis and high fat diets are associated with increased Proteobacteria and Gammaproteobacteria abundance in mice (Cani et al., 2008; Hildebrandt et al., 2009; Reeves et al., 2011). However, despite progressively increasing αSyn levels in the brain, there was no evidence of a pro-inflammatory microbial signature in Thy1-αSyn mice. As other models induce PD symptoms and deficits through environmental triggers (e.g., Rotenone or MPTP treatment) (Johnson et al., 2018; Sun et al., 2018; Yang et al., 2018), or exacerbate PD pathology by oral bacterial administration (Choi et al., 2018) or altering the gut microbiome (Sampson et al., 2016), this suggests that elevated central αSyn levels alone may not drive early intestinal changes in PD. As such, our findings from clinical studies and Thy1-αSyn mice further strengthen evidence for a causative link between microbial dysbiosis and PD progression, supporting a previously reported pathological spread of αSyn from the gut to the CNS in PD pathogenesis (Braak et al., 2006; Hawkes et al., 2007; Tomé et al., 2013; Holmqvist et al., 2014; Stokholm et al., 2016).

As various studies have implicated microbially-derived LPS in PD pathogenesis (Forsyth et al., 2011; He et al., 2013; Kelly et al., 2014; Sharma and Nehru, 2015; Choi et al., 2018), this study was the first to investigate the role of LPS as a catalyst for intestinal permeability and phenotypic change in Thy1-αSyn mice. *In vitro*, a 4-hour LPS treatment noticeably reduced tight junction protein levels (ZO-1 and e-Cadherin) around the cell membrane. Following a 12-day oral LPS treatment, we observed a significant increase in hind-limb clasp score with the tail suspension test in the Thy1-αSyn, but did not observe more severe motor impairments in treated Thy1-αSyn or WT mice, contrary to prior studies utilizing LPS or Thy1-αSyn mice in the context of PD. Previously, an intraperitoneal injection of LPS into healthy C57/BL6 mice resulted in elevations in both normal and aggregated αSyn throughout the large intestine, with a concomitant increase in intestinal permeability, between 3 and 5 months post-LPS treatment (Kelly et al., 2014). Similarly, intranasal administration of LPS to healthy mice every second day for 5 months caused progressive hypokinesia, nigrostriatal αSyn aggregation, selective dopaminergic neuronal loss and accompanying reduction in striatal dopamine, with no evidence of systemic inflammation or immune activation (He et al., 2013). Although these studies demonstrated the marked influence of systemic LPS-induced inflammation on PD pathogenesis in mice, the current study sought to investigate the interaction between a genetic predisposition for PD and intestinal LPS. The failure of Thy1-αSyn mice to develop more severe motor impairments after 12 days of oral LPS treatment may be due to the low dose and less invasive route of administration, which was chosen to mimic more clinically relevant chronic intestinal LPS exposure. As such, future studies in this model should investigate the effects of administering chronic and higher doses of oral LPS, as well as to older mice. Recently, chronic stress exacerbated rotenone-induced PD pathology in mice, increasing intestinal hyper-permeability, markers of oxidative stress, neuroinflammation and fecal *Akkermansia* levels while altering tight junction proteins (Dodiya et al., 2018). Similarly, future studies should characterize the combined influence of different PD risk factors in rodent models, such as environmental toxins and diet.

Microbial dysbiosis may underlie the associations between environmental toxins, pathogens and dietary factors with PD. Rotenone exposure, a Western diet (low fiber/high processed carbohydrate) or high fat diets are linked with dysbiosis, inflammation and PD-like symptoms in rodents (Cani et al., 2008; Hildebrandt et al., 2009; Martinez-Medina et al., 2014; Johnson et al., 2018; Yang et al., 2018), while pesticide and manganese exposure are associated with PD risk (Smyth et al., 1973; Van Der Mark et al., 2011; Kamel et al., 2014). Conversely, exercise, plant-based and Mediterranean diets are associated with a healthy gut microbiome (Tomasello et al., 2016) as well as reduced PD risk and later age of onset (Alcalay et al., 2012), strengthening evidence for environmental modulation of the gut microbiome in PD.

Overall, this study strengthens increasing evidence for a complex interaction between environmental and genetic determinants of PD, where dysbiosis and gastrointestinal dysfunction may act as a catalyst for αSyn pathophysiology and eventual neurodegeneration. Future studies should explore the cumulative risk of various genetic variants involved in immune regulation and intestinal integrity coupled with environmental factors such as nutrition, drug and toxin exposure, and microbial composition. Although a crucial regulator of the gastrointestinal environment, the gut microbiome is easily manipulated, and thus provides an exciting avenue for future clinical interventions to modify the natural course and severity of symptoms in PD.

## Data Availability

There is no dataset.

## Author Contributions

SD, FM, and RA contributed to design of the study. AG, LP, and FK completed *in vivo* characterization. AG, CG, GM, and RA conducted *in vitro* experiments. MB and JK collected and analyzed the clinical patient data. AG and LP performed the statistical analysis. FM, AA, and RA aided with analysis and interpretation. AG and RA wrote the first draft of the manuscript. LP, SD, FM, AA, FK, and RA contributed to critical revision and editing of the manuscript.

## Conflict of Interest Statement

LP and FK are employed by Ozgene Pty Ltd. The remaining authors declare that the research was conducted in the absence of any commercial or financial relationships that could be construed as a potential conflict of interest.
