# Supplementary material for: Altered Gut Microbiome in Parkinson’s Disease and the Influence of Lipopolysaccharide in a Human α-Synuclein Over-Expressing Mouse Model
Source: Front Neurosci. 2019 Aug 7;13:839. doi: 10.3389/fnins.2019.00839 (PMC6693556; doi:10.3389/fnins.2019.00839)
Supplement: Supplementary file 1 [file Data_Sheet_1.docx]

Supplementary Material

# Supplementary Figures and Tables

##
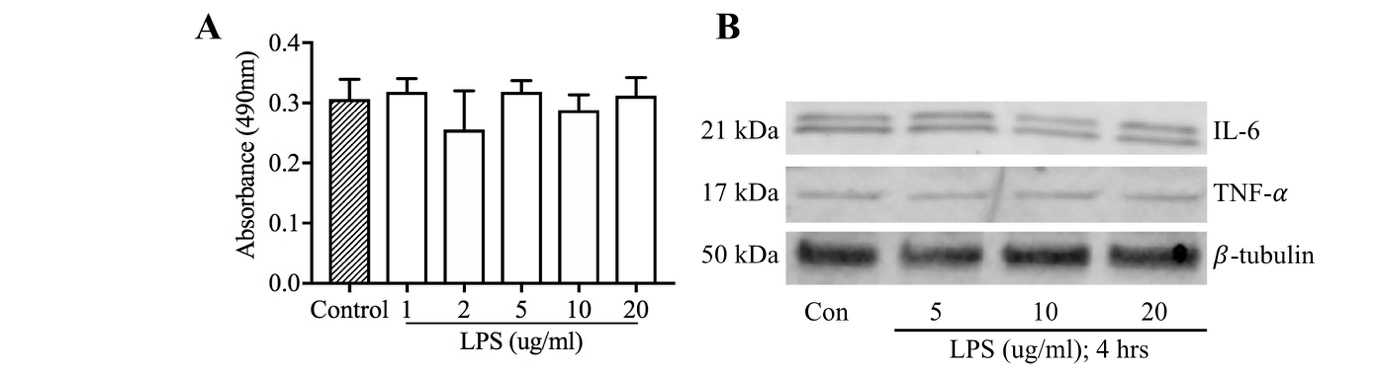
Supplementary Figures

**Supplementary Figure 1. 4-hour lipopolysaccharide treatment did not cause stress in IEC-6 cells.** No significant cell death was observed after 4-hour LPS treatment **(A)**, and Western blots demonstrated no change in inflammatory cytokine levels **(B)**.

**
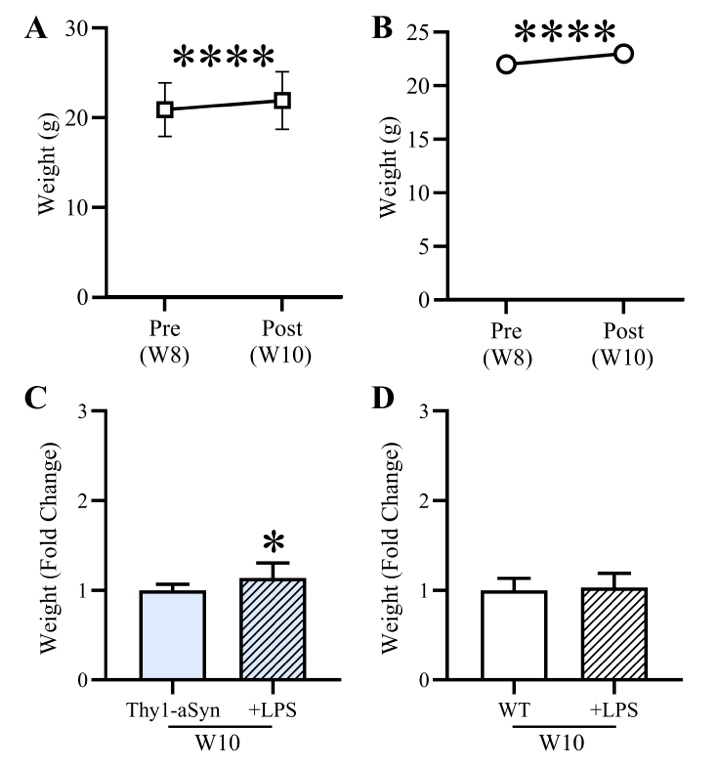
Supplementary Figure 2. LPS treatment caused a significant weight gain compared to baseline in Thy1-αSyn and WT mice.** Thy1-αSyn and WT LPS-treated mice were significantly heavier than baseline **(A & B)**. Treated Thy1-αSyn mice were also significantly heavier than untreated mice at 10 weeks old **(C).** The observed weight gain is a possible consequence of the sucrose in the LPS mixture.

## Supplementary Tables

**Supplementary Table 1.** Clinical characteristics of Parkinson’s disease and control participants.

|  | Control | Mild PD | Severe PD |
| --- | --- | --- | --- |
| *Female (n)* | 3 | 4 | 2 |
| *Age (years)* | 54.7 (16.4) | 62.3 (5.5) | 66.4 (4.4) |
| *PD Duration (years)* | - | 11.1 (3.9) | 15.7 (3.6) * |
| *UPDRS PII* | - | 9.3 (2.8) | 21.3 (7.0) ** |
| *UPDRS PIII* | - | 8.2 (2.9) | 22.4 (14.8) * |
| *PDQ39* | - | 25.6 (13.4) | 59.0 (34.5) * |
| *ACE-R* | - | 94.2 (4.9) | 85.1 (8.0) * |

* *p* < 0.05, ***p* < 0.01, comparison between mild and severe groups. Scores shown as mean (SD). *n* = 7 per group.

**Supplementary Table 2.** Mean mouse weight from 4 - 12 weeks of age.

| Age  (weeks) | Wild Type | | Thy-1 αSyn | |
| --- | --- | --- | --- | --- |
|  | Weight (g) | *n* | Weight (g) | *n* |
| 4 | 18.3 (2.7) | 19 | 16.4 (1.7) | 26 |
| 6 | 20.0 (2.9) | 19 | 18.2 (2.2) | 26 |
| 8 | 21.6 (3.3) | 19 | 19.5 (2.6) | 26 |
| 10 | 23.1 (3.0) | 8 | 19.3 (1.3) | 11 |
| 12 | 24.4 (3.6) | 5 | 20.5 (2.0) | 8 |

Weights shown as mean (SD).
